# Unraveling the Kaposi Sarcoma-Associated Herpesvirus (KSHV) Lifecycle: An Overview of Latency, Lytic Replication, and KSHV-Associated Diseases

**DOI:** 10.3390/v17020177

**Published:** 2025-01-26

**Authors:** Victor A. Losay, Blossom Damania

**Affiliations:** 1 Lineberger Comprehensive Cancer Center, University of North Carolina, Chapel Hill, NC 27599, USA; viclosay@email.unc.edu; 2 Department of Pharmacology, University of North Carolina, Chapel Hill, NC 27599, USA; 3 Department of Microbiology & Immunology, University of North Carolina, Chapel Hill, NC 27599, USA

**Keywords:** Kaposi sarcoma-associated herpesvirus (KSHV), Kaposi sarcoma (KS), primary effusion lymphoma (PEL), latency, LANA, lytic replication, oncogenic virus

## Abstract

Kaposi sarcoma-associated herpesvirus (KSHV) is an oncogenic gammaherpesvirus and the etiological agent of several diseases. These include the malignancies Kaposi sarcoma (KS), primary effusion lymphoma (PEL), and multicentric Castleman disease (MCD), as well as the inflammatory disorder KSHV inflammatory cytokine syndrome (KICS). The KSHV lifecycle is characterized by two phases: a default latent phase and a lytic replication cycle. During latency, the virus persists as an episome within host cells, expressing a limited subset of viral genes to evade immune surveillance while promoting cellular transformation. The lytic phase, triggered by various stimuli, results in the expression of the full viral genome, production of infectious virions, and modulation of the tumor microenvironment. Both phases of the KSHV lifecycle play crucial roles in driving viral pathogenesis, influencing oncogenesis and immune evasion. This review dives into the intricate world of the KSHV lifecycle, focusing on the molecular mechanisms that drive its latent and lytic phases, their roles in disease progression, and current therapeutic strategies.

## 1. Introduction

It is estimated that approximately 15% of cancer cases worldwide are caused by infectious agents [1]. Kaposi sarcoma-associated herpesvirus (KSHV), also known as human herpesvirus 8 (HHV8), is the eighth human herpesvirus discovered, and one of seven viruses known to cause cancer. Genomic analysis further classifies KSHV as a member of the *Gammaherpesvirinae* subfamily and the only human *Rhadinovirus* discovered to date [2,3].

KSHV is like other herpesviruses in its ability to establish lifelong infection in its host. This occurs through a biphasic cycle consisting of quiescent latent and productive lytic phases. Latency is defined by minimal gene expression, which ensures the maintenance of the viral genome. In the latent phase, the viral genome is maintained and replicated as a circular episome by host cell machinery [4]. Various stimuli can induce lytic reactivation and replication. Upon reactivation, viral replication ensues, where viral genes are expressed in a temporal cascade, leading to the production and release of infectious virions [5,6,7].

KSHV was discovered in Kaposi sarcoma (KS) lesions [8] and has since been confirmed as the causative agent of KS, as well as two lymphoproliferative diseases, namely, primary effusion lymphoma (PEL) and multicentric Castleman disease (MCD). KSHV is also associated with an inflammatory condition named KSHV inflammatory cytokine syndrome (KICS) [8,9,10,11]. KSHV-associated malignancies are most often observed in immunocompromised patients such as HIV-infected individuals or transplant patients.

## 2. Virion Structure, Viral Genome, and KSHV Entry

### 2.1. Virion Structure

The KSHV virion is structurally very similar to other herpesviruses, characterized by an icosahedral nucleocapsid surrounded by a protein layer called the tegument, which is surrounded by a lipid bilayer. Cryo-EM and mass spectrometric analysis has shown that the KSHV capsid is composed of six distinct viral proteins: the major capsid protein (MCP), which is encoded by ORF25; the small capsid protein (SCP), which is encoded by ORF65; the triplex 1 and 2 proteins (Tri1 and Tri2), which are encoded by ORF26 and ORF62; the portal protein (PORT), which is encoded by ORF43; and the scaffolding assembly protein (SCAF), which is encoded by ORF17.5 and is not present in the mature C capsid [12,13,14]. The viral envelope is studded with many viral proteins that perform various roles in attachment and entry. These include six glycoproteins: B (gB), H (gH), M, (gM), L (gL), and N (gN). The glycoproteins are encoded by ORF8, ORF22, ORF39, ORF47, ORF53, and a unique KSHV gene named K8.1 [15].

The tegument is a blend of viral proteins and RNAs that contributes to the establishment of viral persistence upon infection. The tegument is an area of active research as its various roles are much more nuanced than other components of the KSHV virion. Significant work has been carried out to determine what makes up the tegument. An earlier study showed that a variety of viral proteins localize to the tegument; these include ORF21 (also known as viral thymidine kinase or vTK), ORF33, ORF45, ORF63, ORF64, and ORF75 [15]. A subsequent study also showed that lytic proteins ORF50 and K8 were present in the tegument [16]. Both studies uncovered the presence of various cellular proteins in the tegument, e.g., tubulin, non-muscle beta-actin, and chaperone protein (heat shock cognate 70). Viral RNAs are also present in the tegument [17]. Once thought to be an unstructured mixture of proteins and RNAs, the tegument is now recognized to be an organized, partially asymmetrical structure with distinct layers and scaffolds [18,19]. Closely associated with the capsid lies the inner tegument, which contains the capsid-associated tegument complexes (CATCs) and helps to stabilize the virion and link the capsid to the outer tegument [20]. The outer tegument surrounds the inner tegument layer and lies near the viral envelope. This layer is less organized and houses proteins that hold roles in virion transportation, signaling, and immune evasion upon viral entry [21].

### 2.2. KSHV Genome

Inside the nucleocapsid lies the KSHV viral genome, a large double-stranded linear DNA genome encoding over 90 open reading frames (ORFs) [5]. The KSHV nucleotide sequence was first obtained by sequencing the BC-1 cell line, an established PEL cell line obtained from effusion samples of a middle-aged man with acquired immunodeficiency syndrome (AIDS) [22]. Sequencing revealed a central 140.5 kb coding region flanked by 801 bp GC-rich terminal repeat (TR) sequences. The ORFs are named in order from left to right, where unique KSHV ORFs are given the letter “K” as a designation. Greater than 60% of ORFs share homology with the various members of the herpesvirus family. The unique ORFs, named K1–K15, encode for more than 15 proteins due to alternative translation initiation sites and splicing. Interestingly, several KSHV ORFs encode for viral homologs of cellular cytokines, viral interferon regulatory factors, anti-apoptotic proteins, and viral homologs of cell cycle proteins. The KSHV genome also encodes for multiple RNAs, which include micro RNAs (miRNAs), long non-coding RNAs (lncRNAs), and circular RNAs. Transcriptional mapping has allowed for the determination of three distinct classes of viral transcription: genes expressed during latency, genes expressed during lytic replication, and a third class of genes expressed at low levels during latency but upregulated during lytic reactivation [23]. Furthermore, two additional studies have deepened our understanding of the complex nature of the KSHV genome, revealing novel genomic and functional features [24,25].

### 2.3. KSHV Entry

KSHV viral entry is mediated by the interactions of the multitude of glycoproteins present on the viral envelope with host cell surface receptors. KSHV has been shown to infect endothelial cells, B cells, epithelial cells, and monocytes in vivo. However, it can infect several other cell types in cell culture. Of note, KSHV can also infect monkey, hamster, and mouse cells in vitro. KSHV entry is mediated by its glycoproteins, namely gB, the gH-gL dimer, and gK8.1. These are essential for successful viral entry. KSHV glycoproteins interact with a multitude of cellular receptors depending on the cell type, and these interactions are described below.

Heparan sulfate proteoglycans (HSPGs) are ubiquitously expressed on the surface of host cells and serve as an important receptor for KSHV target cell recognition. This is demonstrated by the high affinity of various viral glycoproteins for HSPGs, specifically gB, gH, and gpK8.1A [26,27,28]. Although soluble heparan reduced infection, it did not prevent it. KSHV complement control protein (KCP), which is encoded by ORF4, is also able to bind to HSPGs [29]. KSHV gB has an RGD integrin-binding motif, which contributes to binding to target host cells through integrin alpha3beta1 expressed on the cell surface [30,31]. KSHV is also able to bind non-integrin cell surface molecules, namely, dendritic cell-specific intercellular adhesion molecule (ICAM)-3 grabbing nonintegrin (DC-SIGN), which are expressed on macrophages and dendritic cells [32].

Cysteine/glutamate antiporter (xCT), which is found in complex with the cell surface protein CD98 and is expressed on a myriad of cell types (e.g., immune cells, epithelial and stromal cells, etc.), holds a key role in the permissivity of cells to infection. Specifically, KSHV binds to xCT on a variety of cell lines, and the expression of xCT on non-permissible cells allows for infection [33]. KSHV directly upregulates xCT expression via miRNA, KSHV-miR-k12-11 [34]. This miRNA downregulates BACH-1, a known repressor of xCT expression, to increase viral binding and entry as well as protect infected cells from reactive nitrogen species [35]. This points to the critical role of xCT; therefore, it does not come as a surprise that the KSHV-xCT interaction is essential for efficient infection in adherent cells [36].

Finally, other cell surface receptors known to play roles in KSHV binding are the ephrin receptor tyrosine kinases A2 and A4 (EphA2 and EphA4), which are expressed on epithelial, endothelial cells, and fibroblasts. EphA2 and EphA4 specifically interact with KSHV gH-gL dimer, with EphA4 promoting stronger interaction and better entry [37,38]. These receptors, like the xCT interaction, hold a more direct role in binding and entry, as deduced from the finding that prevention of these interactions resulted in inhibition of KSHV infection [39]. Activation of the focal adhesion kinase (FAK) pathway following the binding of KSHV to host cells has been shown to enhance viral entry [30,40]. The FAK pathway is driven by the activation of a tyrosine kinase following integrin signaling.

Receptor-mediated endocytosis, driven by interactions of the virus with receptors like DC-SIGN, xCT, and ephrin, is believed to be the dominant form of KSHV viral entry. Following endocytosis, the capsid uses cytoskeletal machinery to traffic to the nucleus and release viral DNA.

## 3. Latency: Quiescent Phase of the Lifecycle

### 3.1. Establishment of Latency

Following primary infection, KSHV can enter into either a persistent latent or productive lytic phase of replication. Numerous studies performed in vitro have shown that latency is the preferred pathway for KSHV infection in cell culture. Studies in endothelial and fibroblast cells have shown that primary infection results in the initial expression of both latent and lytic genes [41]. Specifically, early expression of both replication and transcription activator (RTA) and latency-associated nuclear antigen (LANA) was observed. However, the expression of RTA and some other lytic genes decreased, eventually falling below detection levels, while latency prevailed. During latency, gene expression is limited, and the viral genome is tethered to the host chromosome and replicated using the host cell machinery during cell division. Furthermore, no virus is produced during latency, but KSHV persists in cellular reservoirs like B cells and endothelial cells.

The classical latency program is defined by the expression of a select few genes originating from a single locus located near the 120 kb region in the viral genome. Two active latent promoters are present in that region, LANA and Kaposin promoters, known as LTc and LTd, respectively [42]. The LTc promoter generates three RNAs, which are responsible for the expression of ORF73, ORF72, and ORF71, known as LANA, vCyclin, and vFLIP, respectively [43,44,45]. The three RNAs share the same 3′ coterminal but are initiated at different 5′ sites. The LTd promoter is located downstream of ORF73 and drives the expression of different ORF72 and ORF71 transcripts as well as the K12 transcript, which generates all three Kaposin proteins (A, B, and C) [46,47]. A third inducible promoter known to generate transcripts from the primary latent locus was discovered based on an observation that showed increased LANA levels following RTA induction [48]. This third promoter, LTi, is located just downstream of LTc and is activated by the expression of RTA [49,50]. Four additional genes, v-IRF3, K1, K2, and K15, are transcribed during latency [51,52,53], along with multiple viral miRNAs [54,55].

### 3.2. KSHV Latency-Associated Nuclear Antigen (LANA)

**Figure 1:**
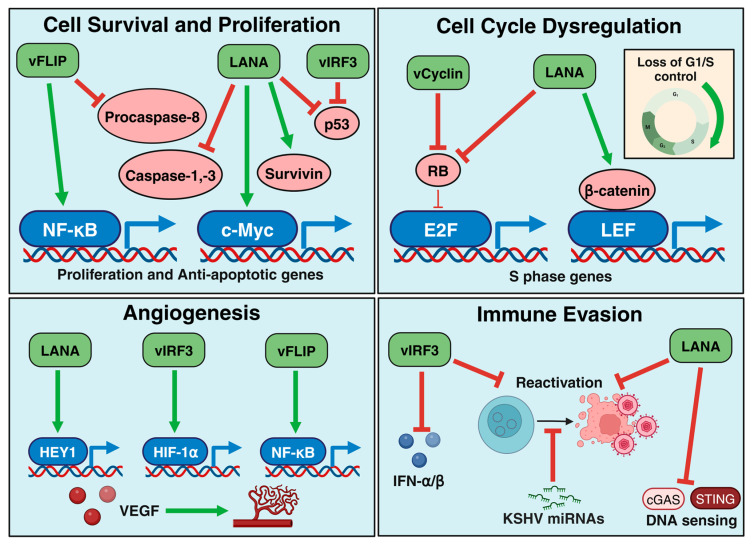
Host cell transformation by KSHV latent proteins. The KSHV latent proteins (LANA, vFLIP, vCyclin, and vIRF3) promote cell survival and proliferation, cell cycle dysregulation, angiogenesis, and immune evasion.

LANA is a complex protein that lies at the heart of latency and is defined as one of the most important viral proteins due to its many roles (Figure 1). LANA is a large nuclear protein expressed in all KS lesions and KSHV-infected PEL patient samples [56]. It is encoded by ORF73 and is stably expressed during viral latency [57,58]. Historically, LANA has been used as a serologic marker for KSHV infection in patients. In fact, “LANA speckles” in the nuclei of infected cells are a hallmark of KSHV latency.

LANA tethers the KSHV episome to cellular DNA, ensuring the maintenance of the viral genome [59,60]. LANA is made up of three structurally distinct domains: a highly acidic core flanked by DNA-interacting N- and C- termini, each with unique functions. The C-terminal domain recognizes specific viral DNA terminal repeats (TRs) and allows for viral episome persistence in the host [61]. The three specific sites on the TRs are called LANA binding sites 1 (LBS1), 2 (LBS2), and 3 (LBS3), where LBS3 is a GC-rich replication element. In a manner that is reminiscent of Epstein–Barr virus nuclear antigen 1 (EBNA1)’s role in the maintenance of the EBV genome, LANA binds multiple specific TR sites, allowing for transcriptional and replicative control over the viral genome [62,63]. The N-terminal domain targets LANA to the nucleus via its nuclear localization signal (NLS), and following entry into the nucleus, it interacts with cellular histones and tethers the viral genome to host DNA [64]. The structural ability of the N-terminal domain to attach to mitotic chromosomes is what allows for the maintenance of the viral episome via segregation to both daughter cells. Super-resolution imaging (dSTORM) has allowed for a clearer understanding of the tethering machinery, showing the binding of the N- and C- termini to genomic and viral DNA, respectively [65]. By tethering, LANA promotes the recruitment of cellular replication machinery, which includes proteins like origin recognition complex (ORC), minichromosome maintenance (MCM) proteins, replication factor C (RFC), and DNA polymerase clamp loader [66,67,68]. This recruitment allows LANA to initiate semiconservative viral DNA replication using host cell machinery once per cell cycle [69,70,71]. However, it is important to note that latent viral replication has also been reported to occur in a LANA-independent manner [72].

In addition, LANA has been shown to regulate both host and viral gene transcription [73,74]. Most of this transcription regulation is driven by the core domain of LANA. The core domain of LANA consists of acidic amino acid repeats, which confer a “sticky” property that enhances LANA’s ability to bind to other proteins and provides the structural flexibility needed to accommodate its various binding partners.

Specifically, LANA binds to the tumor suppressor p53, downregulating its transcriptional activity [75]. LANA upregulates and induces activation of the pro-survival protein survivin, which promotes cell survival as well as regulates viral replication through inhibition of histone deacetylase (HDAC) [76]. LANA impacts the notch signaling pathway through the stabilization of a downstream effector, hairy/enhancer-of-split related with YRPW motif protein 1 (Hey1), by preventing degradation and promoting angiogenesis [77]. LANA binds Rb and prevents its inhibition of early region 2 binding factor (E2F) transcription factors, resulting in increased expression of DNA replication and cell cycle progression genes [78]. LANA increases the protein expression of beta-catenin by binding to the kinase glycogen synthase kinase-3 beta (GSK-3b), and this interaction prevents ubiquitin-dependent degradation of beta-catenin. As a result, beta-catenin accumulation affects the transcription of proliferation genes through its interaction with lymphoid enhancer-binding factor (LEF), which stimulates entry into the S phase [79]. This same interaction of LANA with GSK-3b also results in the stabilization and activation of the oncoprotein c-Myc, another transcription factor with pro-survival and proliferation properties [80,81]. LANA induces aneuploidy by disrupting the actions of shugoshin-1 (Sgo1), a protein with a role in protecting the integrity of centromeres. This chromosomal instability specifically results from LANA’s interaction with mitotic checkpoint kinase budding uninhibited by benzimidazoles 1 (Bub1), which inhibits Bub1-mediated phosphorylation of H2A and results in the dislocation of Sgo1 from the centromeres [68,82].

LANA represses lytic gene transcription by interacting with notch signaling. This occurs through binding with recombination signal binding protein for immunoglobulin kappa J region (RBPJ, also known as RBP-jK), which inhibits ICN-mediated transactivation of RTA [83,84]. LANA also binds proteins with roles in epigenetic control and chromatin regulation, such as BRD2, HP1, and hSET1 [85,86,87]. Through these interactions, LANA can manipulate host and viral gene expression. LANA also promotes cell survival by preventing apoptosis and inflammasome activation, mediated by interaction with caspases [88]. Finally, the cytoplasmic version of LANA can inhibit cyclic GMP-AMP synthase (cGAS)-stimulator of interferon genes (STING)-mediated DNA sensing in the cytoplasm [89,90,91]. Through these various interactions, LANA mediates the maintenance of the viral episome and promotes both cell proliferation and survival.

### 3.3. KSHV Viral Cyclin (vCyclin)

Another latent protein encoded by KSHV ORF72 is vCyclin, a homolog of cellular cyclin D [92]. vCyclin has a role in cell cycle regulation and cell survival. Specifically, vCyclin can bind and activate various cyclin-dependent kinases (CDKs) like CDK6 and CDK4, although it has a stronger affinity for CDK6 [93]. This activation of CDK6 has been shown to specifically phosphorylate various cellular targets like Rb, histone H1, and nucleophosmin, impacting cell cycle progression into the S phase as well as transcriptional control [93,94]. Furthermore, vCyclin-induced activation of CDK6 is resistant to CDK inhibitors [95]. It has also been demonstrated that vCyclin overrides contact inhibition to promote cell growth and tumorigenesis [96].

vCyclin is unique as it can exhibit a dual role, with both beneficial and deleterious effects in cells. This duality is best exemplified by its potential to cause apoptosis [97]. vCyclin-induced apoptosis results from p53 activation and bcl-2 inactivation, driven by CDK6-mediated phosphorylation [98,99]. p53 activation can also occur through a different mechanism, mediated by vCyclin activation of CDK9 [100]. Furthermore, vCyclin also induces senescence and the DNA damage response in vitro [101]. It is worth noting that this p53-mediated apoptosis can be controlled by other latent proteins, like LANA, as mentioned above through the inhibition of p53.

### 3.4. KSHV Viral FLICE-Inhibitory Protein (vFLIP)

Another latent gene is a viral homolog of cellular FLICE-inhibitory protein (FLIP) called vFLIP. vFLIP is encoded by ORF71. vFLIP shares structural homology with the short form of cellular FLIP (FLIPs) and can also block the apoptotic cell death program [102]. Specifically, vFLIP prevents Fas-dependent activation of caspase 8 by binding and inhibiting procaspase-8 [103]. However, it is important to note that some groups have found that vFLIP-expressing mice are not resistant to Fas-mediated apoptosis [104].

vFLIP can strongly activate the nuclear factor-kappa B (NF-κB) pathway, a family of pro-survival transcription factors [105]. Specifically, vFLIP modulates the activity of the NF-κB pathway through physical interaction with proteins upstream of the pathway or directly involved in its activation, like tumor necrosis factor receptor-associated factor (TRAF) and NF-kappa-B essential modulator (NEMO) [106,107]. Both the canonical and noncanonical NF-κB pathways are activated by vFLIP [108]. Activation of these pathways has been shown to have transforming abilities both in vitro and in vivo, highlighting the importance of this protein in oncogenesis [104,109,110]. This key role in KSHV-infected cells is further exemplified by the vulnerability of KSHV-infected cells to NF-κB inhibition. Chemical or genetic inhibition of NF-κB signaling induces apoptosis in viral cancers in both in vitro and in vivo studies, showcasing the essential role of vFLIP [111,112].

### 3.5. KSHV Kaposins

The Kaposin proteins are encoded by a unique KSHV ORF named K12 and are expressed at low levels during latency and upregulated during lytic replication [113]. Alternative splicing and variable inclusion of upstream direct repeat sequences (DR1 and DR2) drive the complex translational machinery that results in the formation of multiple Kaposin proteins. Kaposin A is translated without the DR sequences solely from the K12 locus; however, Kaposins B and C are translated from different CUG start codons upstream of the DR sequences. Kaposin B is unique in that it does not include translation of the K12 sequence, a highly hydrophobic section that binds the membrane. Subcellular localization studies have shown that this unique translation machinery allows for varying compartmentalization of the Kaposin proteins [114].

In vivo studies have shown that Kaposin A has transforming capabilities [115], partly due to Kaposin A’s interaction with cytohesin-1. Specifically, Kaposin A stimulates cytohesin-1, a guanine nucleotide exchange factor (GEF), to activate ARF1, a GTPase with various cellular functions [116]. Kaposin B activates the p38 MAPK and MK2 kinase pathway, resulting in decreased degradation and increased stability of pro-inflammatory cytokine mRNAs [117]. This action is dependent on the DR1 and DR2 repeats that Kaposin B expresses [118]. Kaposin B also improves PROX1 mRNA stability, resulting in accumulation of protein in the cytoplasm. This protein has a role in the lymphatic reprogramming of vascular endothelial cells, which might explain some of the transformation observed in KS tumor cells [119]. Very little is known about the role of Kaposin C, although structural similarities with the other Kaposin proteins point to complementary roles.

### 3.6. LANA2/Viral Interferon Regulatory Factor 3 (vIRF3)

ORF K10.5 encodes one of four viral interferon regulatory factor (IRF) homologs that KSHV expresses and is named viral IRF-3 (vIRF3) [120]. It was originally named LANA2 due to its latent expression in PEL cells [121]. Analysis of vIRF3 expression in PEL revealed that expression levels are increased following lytic reactivation and that vIRF3 localizes to the nucleus of infected cells [51].

vIRF3 inhibits specific post-translational modifications of p53, which govern its activity, such as inhibiting sumoylation of p53 by small ubiquitin-like modifier 2 (SUMO2) [122]. As a result, vIRF3 inhibits p53-induced transcription. vIRF3 also inhibits the expression of type I interferon (IFN-α/β) genes [120]. vIRF3 can directly bind to IRF7, preventing its association with DNA and preventing it from activating the expression of IFN-alpha [123]. In a similar preventative fashion, vIRF3 has also been found to inhibit the transcriptional activity of IRF5 [124]. This provides another advantage for viral persistence as IRF5 induction in normal conditions has been shown to result in interferon-mediated apoptosis and cell cycle arrest. vIRF3 also helps in controlling both PEL cell proliferation and the KSHV life cycle through its interaction with ubiquitin-specific peptidase 7 (USP7) [125]. Wies et al. found that expression of vIRF3 is essential for survival of PEL cells, as knockdown of vIRF3 resulted in increased caspase activity and reduced proliferation and survival [126].

vIRF3 is not expressed in KS spindle cells [121]; however, it is expressed in endothelial cells with a novel role in stabilizing hypoxia-inducible factor-1 (HIF-1), a protein with a role in angiogenesis [127]. Specifically, vIRF3 interacts with and stabilizes the HIF-1 alpha subunit, which drives vascular endothelial growth factor (VEGF) expression and promotes the formation of new blood vessels to provide the tumor with nutrients. Furthermore, vIRF3 interacts with HDAC5, altering gene expression to promote lymphangiogenesis and the hypersprouting of lymphatic endothelial cells (LECs) [128].

### 3.7. microRNAs: Tiny Regulators, Big Impact

KSHV encodes 12 pre-microRNAs (pre-miRNAs) that arise from the primary latency locus [54,129,130,131]. miR-K1-K9 are clustered together while miR-K10 and miR-K12 are located downstream. These pre-miRNAs can mature into 25 miRNAs following cleavage by endonucleases Drosha and Dicer and then eventually assemble into an RNA-induced silencing complex (RISC), which allows them to target mRNAs. These miRNAs are latently expressed in both PEL and KS [132]. Studies have shown that numerous cellular genes are affected by these miRNAs, including genes involved in cell proliferation and cell survival [133,134]. miRNAs contribute to the transformation of KSHV-infected endothelial cells by reprogramming transcription [135]. The viral miRNAs also hold a key role in regulating the viral life cycle, specifically in promoting latency and preventing lytic reactivation [136,137,138,139]. Studies have also shown that miRNAs can be packaged into exosomes (extracellular vesicles) and exported out of cells, therefore impacting neighboring cells [140,141].

More recently, circular RNAs (circRNAs) were found in KSHV-infected cells [142,143,144]. These can inhibit other RNAs or interact with RNA-binding proteins. The circRNAs are encoded within ORFs of lytic genes that are expressed during lytic replication. Additionally, one study revealed that KSHV induces the expression of a human circRNA (hsa_circ_0001400), which plays a role in promoting latency following infection [145].

## 4. KSHV Signaling Proteins

Herpesviruses are known for their biphasic cycle. However, some genes can be expressed during both latency and lytic replication. Specifically, three viral genes (K1, K2, and K15) are expressed at low levels in latency and upregulated during lytic reactivation [52,146,147]. All three are potent signaling proteins, so their low expression during latency is most likely enough to achieve their designated roles.

### 4.1. KSHV K1

K1 is a type I transmembrane protein that can be found on the plasma membrane as well as in the ER. K1 has a domain that protrudes out into the extracellular space, and it has a cytoplasmic domain that includes an immunoreceptor tyrosine-based activation motif (ITAM) [148]. These domains allow K1 to signal in a way similar to the B cell antigen receptor (BCR), where activation results in phosphorylation of tyrosines, leads to the downstream activation of proliferative and survival pathways like the phosphoinositide 3 kinase (PI3K)/Akt/mammalian target of rapamycin (mTOR) and adenosine monophosphate-activated protein kinase (AMPK) pathways, and induces inflammatory cytokine production [149,150,151]. K1 also promotes cell survival by interacting with Hsp90beta and Hsp40 [152]. Studies have shown that K1 is constitutively active and does not require ligand binding to promote its effects on infected cells [153].

Furthermore, K1 has transforming abilities, as shown in various studies, with a particular role in angiogenesis and tumorigenesis [154,155,156,157]. Finally, K1 has been shown to have a dual effect on the viral life cycle depending on the cell type, with a reciprocal role in augmenting and suppressing lytic replication in an ITAM-dependent manner, demonstrating the complexity of this protein [158,159,160].

### 4.2. KSHV K2/vIL-6

K2 encodes for a viral homolog of cellular human IL-6 (hIL-6) known as vIL-6, which is a signaling cytokine involved in immune response regulation and inflammation, which binds to a receptor complex to allow cellular changes. vIL-6 differs from its cellular counterpart in that it does not require a co-receptor to activate downstream signaling pathways, such as the activation of signal transducer and activator of transcription 1 and 3 (STAT1 and STAT3) [161]. Although vIL-6 is less potent than IL-6, its ability to signal without a co-receptor allows it to affect a larger number of cells. In the context of KSHV-associated diseases, vIL-6 holds transformative properties, particularly promoting angiogenesis, endothelial cell migration [162], hematopoiesis [163] and plasmacytosis [164] in various in vitro and in vivo models. vIL-6 also induces an increase in VEGF, angiopoietin 2, and integrin β3 [163,165,166]. Finally, vIL-6 has also been implicated in B-cell survival and the pathogenesis of PEL and MCD. Certain studies have shown that vIL-6 has anti-apoptotic properties in PEL, and the knockdown of vIL-6 greatly reduced cell proliferation [167,168].

### 4.3. KSHV K15

K15 was originally named latency-associated membrane protein (LAMP) [169]. K15 expression results in multiple protein variants with a common cytosolic tail. These variants localize to the intracellular and plasma membranes.

The K15 cytoplasmic tail is highly dynamic and can be tyrosine phosphorylated. This phosphorylation has been shown to specifically prevent BCR signaling [170]. In addition, the cytoplasmic tail contains a TRAF-binding site, which is involved in the activation of various signaling pathways including Ras/MAPK, NF-kB, and c-Jun N-terminal kinase (JNK) [171]. K15 also modulates extracellular crosstalk by inducing the expression of activator protein-1 (AP-1) and pro-inflammatory cytokines like IL-6, IL-8, and Dscr1 [172]. K15 also modulates cellular immunity by interacting with members of the Src family kinases (SFKs) [173]. K15 has a direct anti-apoptotic role through its interaction with HS1-associated protein X-1 (HAX-1) [53]. Finally, K15 plays a role in promoting angiogenesis, and its expression in KS lesions provides a rationale for this [174]. Interestingly, both K15 and K1 are critical for productive lytic replication [147].

## 5. Lytic Replication

As mentioned above, latency appears to be the default pathway for KSHV infection in cell culture. However, various factors were found to induce lytic reactivation. These factors include but are not limited to ER and oxidative stress, and hypoxia. Following lytic reactivation, the cascade of lytic replication occurs, defined by the temporal expression of immediate early (IE) and delayed early (DE) proteins. This is followed by complete lytic viral replication, the expression of late viral proteins and the eventual formation and release of infectious virions. Of note, abortive lytic replication has been found to occur where expression of some lytic genes occurs, but there is no production of virion progeny.

### 5.1. KSHV RTA: Master Regulator of the Lytic Switch

RTA, which is encoded by ORF50, is an immediate early protein and the key lytic switch protein. Certain studies have proved that RTA expression is necessary and sufficient for lytic reactivation and RTA silencing prevents lytic reactivation [175,176,177,178]. RTA’s primary role is as a viral transcription factor, and this functionality is highly conserved across the Rhadinovirus family members [179]. RTA is made up of an N-terminal DNA-binding domain (DBD) and a C-terminal activation domain, which can drive transcription following phosphorylation [177]. It has been shown that the transcription activity is highly dependent on RTA assembling into a tetramer [180].

RTA binds to a multitude of sites on the viral genome, including but not limited to promoters of lytic genes in addition to its promoter, LANA, as well as both origins of lytic replication (OriLyt-L and OriLyt-R) [181]. RTA has been shown to potently activate the noncoding polyadenylated nuclear (PAN) RNA promoter [182,183,184]. RTA also activates promoters through protein–protein interactions, notably by activating the cellular transcription factor RBPJ [185]. RTA binds to other cellular transcription factors with roles in both activation and suppression of gene expression, namely, with C/EBPalpha, Oct-1, and STAT3 [186,187,188]. To further regulate transcription, RTA interacts with histone acetylases and chromatin remodeling complexes (SWI/SNF) [189,190]. RTA also binds proteins with transcription repressing roles, like poly (ADP-ribose) polymerase 1 (PARP-1), KSHV RTA binding protein (K-RBP) transducing-like enhancer of split 2 (TLE2), and inhibitor of DNA binding protein 2 (ID2) [191,192,193,194].

RTA facilitates the degradation of specific proteins through interactions with ubiquitination machinery and ubiquitin ligases. Through these interactions, RTA targets a myriad of viral and cellular proteins for degradation. Notably, RTA prevents the antiviral immune response by directly targeting IRF7 for degradation, thereby blocking both Toll-like receptors 3 and 4 (TLR3 and TLR4) signaling pathways [195,196]. Additionally, RTA degrades Myeloid differentiation factor 88 (MyD88), another protein involved in immune signaling [197]. RTA promotes lytic replication by targeting various proteins, including the transcriptional repressor protein Hey1 [198], vFLIP [199], the protein structural maintenance of chromosome (SMC) complex SMC5/6 [200], and the E3 ubiquitin ligase TRIM32 [201]. A recent ubiquitin-modified proteome analysis further expanded the list of RTA targets, adding the immune surveillance protein HLA-C and cell cycle modulators such as cyclin-dependent kinase 1 (CDK1), minichromosome maintenance 7 (MCM7), and SUMO2/3 [202].

### 5.2. KSHV K8

ORF K8, also known as replication-associated protein (RAP), is another IE protein. It is also named K-bZIP due to its basic leucine zipper (bZIP) domain, which allows for transcriptional regulation. Although K8 has been described as a DE gene, it was discovered via screening as an immediate-early transcript [203], as a result of having two promoters activated in a time-dependent manner [204]. K8 holds a key role in inducing host cell cycle arrest by binding to CCAAT/enhancer binding protein alpha (C/EBPα) and p21, which promotes their stability and forces cells to arrest in the G1 phase [186,205]. This is a conserved herpesvirus mechanism that allows the virus to replicate its genome without the cellular genome competing for host machinery.

On the other hand, K8 can act in a transcriptional repressive fashion, specifically by interacting with the transcription factor CREB-binding protein (CBP) and by recruiting Ubc9 to specific viral promoters [206,207]. K8 also interacts with RTA to modulate both cellular and viral gene expression, showcasing its wide role in modulating lytic replication by repressing and activating various genes [208,209]. K8 also possesses RNA binding capacity and RNA mediates the interaction of K8 and various promoters [210].

### 5.3. KSHV ORF57, Delayed Early Protein

Following the expression of IE genes, DE genes are expressed. DE genes are directly regulated by the expression of IE genes and their main goal is to prepare the infected cell for complete viral replication. One key DE protein is ORF57, also known as mRNA transcript accumulation (MTA). ORF57 is a posttranscriptional regulator of viral transcripts, allowing for the accumulation of mRNAs in the cytoplasm and nucleus [211]. ORF57 interacts with and recruits various cellular complexes to promote mRNA transport, function as a splicing factor, initiate translation of intronless RNAs, and prevent cellular RNA decay [212,213,214,215,216]. Furthermore, ORF57 interacts with RTA to promote the activation of lytic promoters, thus augmenting productive lytic viral replication [217]. These key roles of ORF57 are further highlighted by studies that have shown that disruption of ORF57 expression results in unproductive lytic replication lacking virion production [218].

### 5.4. Viral DNA Replication and Late Gene Expression

Following the expression of DE genes, full viral lytic replication occurs via rolling circle replication using replication complexes that the virus encodes. These include a viral DNA polymerase, helicase, polymerase processivity factor, primase, primase-associated factor, and single-strand binding protein, which are encoded by ORF9, ORF44, ORF59, ORF56, ORF40/41, and ORF6, respectively [219]. Rolling circle replication begins at one of the two nearly identical replication origin sites, OriLyt-R or OriLyt-L [220]. Critical elements required for OriLyt function include AT-rich elements, AP1 transcription factor-binding sites, an ORF50 binding site, a TATA box motif, and a 32 bp sequence [221,222].

Viral DNA replication results in long concatemeric DNA. These linked repeated units of the viral genome are then cleaved into individual linear genomes. Studies in related herpesviruses and KSHV have helped to decipher how viral genome cleavage occurs. It is understood to be driven by the terminase complex, which is comprised of KSHV ORF7, ORF29, and ORF67.5 [223]. Simultaneously, late gene expression drives the production of structural proteins, which perform capsid formation and genome packaging. These include the capsid proteins, as well as portal proteins, which are involved in the opening of the capsid to facilitate genome entry [4,224]. The completed capsid is then packaged with the cleaved linear genome in a process that involves a capsid-associated tegument complex, comprised of ORF19, ORF32, and ORF64, and the portal protein (ORF43) [18,20]. After the viral DNA is packaged, the capsid is sealed and enveloped in a lipid membrane derived from the host. This final step marks the culmination of lytic replication, resulting in the production of infectious virions that are ready to egress.

## 6. Modulation of Cell Pathways by Lytic Proteins

### 6.1. K3 and K5: Modulators of Immune Recognition

K3 and K5, also known as modulators of immune recognition (MIR1 and MIR2), are two highly homologous transmembrane ubiquitin E3 ligases that contain a cytoplasmic-oriented RING finger, which allows for interactions with E2 ubiquitin conjugases [225].

**Figure 2:**
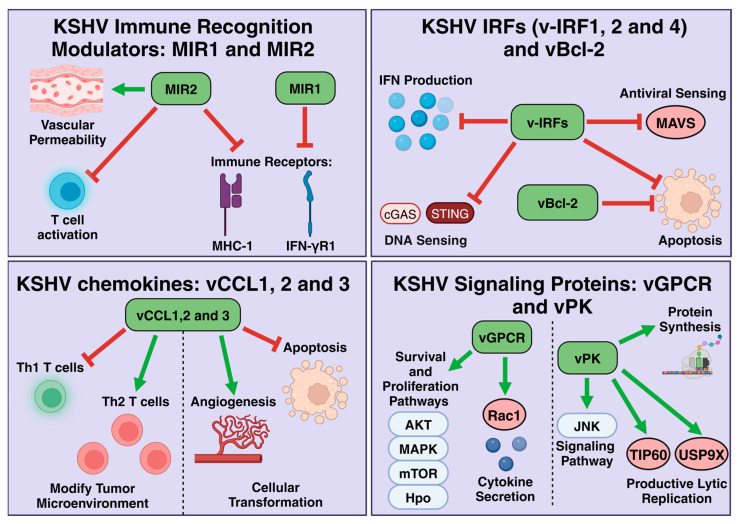
Modulation of cell pathways by KSHV lytic proteins. The KSHV lytic proteins (MIR1 and MIR2, vIRF1, 2, and 4, vCCL1, 2, and 3, vGPCR, vPK, and vBcl-2) manipulate host cells during lytic replication by promoting cell survival pathways, angiogenesis, and vascular permeability and downregulating both the innate and adaptive immune responses.

Through interactions with their RING finger, MIR1 and MIR2 can prevent major histocompatibility complex class I (MHC-1) antigen presentation (Figure 2), with MIR1 and MIR2 having an individual affinity for different human leukocyte antigen (HLA) genes [226,227]. The resulting MIR1- and MIR2-mediated polyubiquitination results in endocytosis and endolysosomal degradation of the MHC-1 proteins [228]. The MIR proteins also downregulate other proteins involved in antigen presentation, such as interferon-gamma receptor 1 (IFN-gammaR1) [229].

In addition to these shared roles, MIR2 holds unique functions in infected cells. Specifically, MIR2 can downregulate the expression of ICAM-1 and B7-2, resulting in decreased T-cell activation [230,231]. MIR2 can also promote vascular permeability, a hallmark of KS, by promoting endocytosis and degradation of vascular endothelial (VE)-cadherin [232]. Finally, the two MIR proteins also differ in the timing of their activity. MIR2 activity is observed initially following lytic phase entry, while MIR1 activity peaks in the later stages of lytic replication [233]. The unique additional roles of MIR2 provide a possible explanation for its earlier activity, as the virus is more vulnerable to immune recognition during early lytic replication.

### 6.2. KSHV IRFs: v-IRF1, 2, and 4

KSHV encodes four viral homologs of cellular IRFs, namely vIRF1-4. vIRF3 is a latent gene described above, while the other three vIRFs are expressed during the lytic cycle. The vIRFs play a key role in antagonizing the functions of cellular IRFs, which results in the suppression of IFN production. vIRF1 is encoded by ORF K9. vIRF1 can inhibit the interferon signaling pathway by directly binding and repressing the actions of the CBP/p300 activators of the interferon antiviral response [234,235,236]. vIRF1 modulates the IFN response by downregulating ISG15 conjugation of IFN-activated proteins [237]. vIRF1 has also been found to inhibit innate immunity by disrupting the cGAS-STING pathway and to promote cell survival by blocking mitochondrial antiviral signaling protein (MAVS) [238,239]. vIRF2 and vIRF4, which are encoded by ORF K10 and K11, have been less extensively studied, but still hold key roles in downregulating both innate and adaptive immunity. vIRF2 prevents IFN-mediated gene transcription by inhibiting IFN-alpha and -beta signaling [240], while vIRF4 downregulates both IRF4- and IRF7-mediated IFN responses [241,242]. In addition to the inhibition of the IFN response in infected cells, the vIRFs also prevent apoptosis by translocating pro-apoptotic proteins and inhibiting p53 [243,244]. A more detailed analysis of the various roles of the vIRFs is described in [245].

### 6.3. Viral Chemokines: vCCL1, 2, and 3

KSHV encodes three CC chemokines known as vCCL1, vCCL2, and vCCL3, which are encoded by ORF K6, K4, and K4.1. These viral chemokines are known for having a role in mediating inflammation and immune evasion [246]. vCCL1-3, also known as viral macrophage inflammatory protein-I-III (vMIP-I-III), has been shown to interact with the cellular chemokine receptors CCR3, CCR4, and CCR8, which are preferentially expressed on T helper type 2 (Th2) cells.

Th1 and Th2 are two subsets of CD4+ T cells that have important roles in orchestrating an immune response. Th1 cells are essential in combatting viral infections as they are the coordinators of cellular immunity, playing a key role in activating immune cells (i.e., cytotoxic T cells and macrophages) to kill infected cells. Th2 cells, on the other hand, play a supporting role in humoral immunity by promoting B-cell activation and the production of IgE antibodies, which are much less effective against intracellular pathogens like KSHV. In the context of KSHV infection, the vCCL proteins preferentially sequester more of the less effective Th2 cells through their specific interactions with Th2-specific receptors. This results in a modified tumor microenvironment that is void of the inflammatory Th1 cells and characterized by a dampened T-cell response [247,248]. The vCCLs also promote angiogenesis in a VEGF-dependent manner and they enhance cell survival by downregulating the expression of the pro-apoptotic protein, Bim [249,250].

### 6.4. Lytic Signaling Proteins: vGPCR and vPK

Viral G-protein coupled receptor (vGPCR) is encoded by ORF74 and is expressed in an ORF50-dependent manner solely during the lytic cycle. vGPCR is unlike cellular GPCRs because it does not require a ligand for activation but is constitutively active [251]. However, studies have shown that cytokines like IL-8 can further activate the signaling capabilities of vGPCR [252]. vGPCR induces cell survival, proliferation, and angiogenesis by stimulating pathways such as the Akt/MAPK/mTOR pathway, inducing the expression of VEGF [253,254,255,256]. vGPCR also specifically activates the small G protein Rac1, resulting in cytokine secretion [257]. Finally, vGPCR modulates the Hippo tumor suppressor pathway, leading to the activation of oncoproteins YAP and TAZ [258]. All these actions of vGPCR highlight the key role that it holds in promoting tumorigenesis and lytic replication [259,260].

Viral protein kinase (vPK) is a hypoxia-induced serine–threonine kinase encoded by ORF36 [261]. vPK localizes to both the nucleus and cytoplasm, activates the JNK pathway, and modulates the DNA damage response [262,263]. vPK was also found to modulate and promote protein synthesis by acting in the same manner as cellular protein S6 kinase (S6KB1) [264]. Finally, vPK also binds cellular ubiquitin-specific peptidase 9X-linked (USP9X) and this interaction is critical for productive lytic replication [265]. The transformative role of vPK is further highlighted by its ability to promote lymphomagenesis in a mouse study [266].

### 6.5. KSHV vBcl-2

Viral B-cell lymphoma-2 (vBcl-2), encoded by ORF16, is transcribed during lytic replication of KSHV [267]. It shares homology with the cellular protein Bcl-2, and its expression has been shown to inhibit apoptosis and autophagy, promoting the survival of the infected cell [268]. Notably, vBcl-2 does not heterodimerize with the pro-apoptotic proteins Bax or Bak, suggesting an evolved mechanism that allows resistance to host apoptotic control. Although vBcl-2 is expressed at low levels during the lytic cycle, it plays a key role in facilitating efficient reactivation from latency [269]. Interestingly, two separate studies demonstrated that vBcl-2 played an essential role in producing infectious virions but this appeared not to be dependent on the anti-apoptotic or anti-autophagy functions of vBcl-2, suggesting the existence of another novel role for vBcl-2 [270,271].

### 6.6. Contribution of Lytic Proteins to KSHV Pathogenesis

Although not directly involved in replication, the above KSHV lytic proteins play critical roles in promoting viral replication and persistence. Immune modulatory proteins like MIR1/2 and v-IRF1, 2, and 4 silence host defenses by downregulating MHC-1, preventing immune sensing of the virus, and inhibiting interferon signaling [272]. Viral chemokines vCCL1, 2, and 3 skew the immune response by recruiting Th2 cells and suppressing Th1 cells in KS lesions, thus promoting tumor growth and immune evasion [246]. Signaling proteins like vGPCR and vPK activate host signaling pathways, promoting cell proliferation and survival through the release of cytokines like VEGF [273]. The anti-apoptotic and autophagy protein vBcl-2 prevents premature cell death and ensures the production of infectious virions through a novel role not related to its inhibition of apoptosis or autophagy. Collectively, these viral lytic proteins promote host cell survival and enable KSHV replication and the dissemination of virion progeny, thereby contributing to KSHV pathogenesis.

## 7. KSHV-Associated Diseases

### 7.1. Kaposi Sarcoma (KS)

KS is the most prominent form of KSHV-associated disease and is defined as an endothelial cancer that can develop in different areas of the body. KS has a variety of clinical presentations and can be classified into multiple subtypes [274,275]. Classic KS was the first form of KS that was originally discovered. Described by Moritz Kaposi in 1884 as a hemorrhagic sarcoma, it is known as a rare, indolent, and rarely disseminated form that is mostly found in elderly Mediterranean and Eastern European men. Endemic KS was the second form of KS described; it is histologically like Classic KS but occurs in regions of sub-Saharan Africa and parts of China (Xinjiang province) where KSHV is endemic [3,276]. Endemic KS is more aggressive and can also affect children. This form is observed in both sexes and in the context of both HIV+ and HIV− patients. The third form is epidemic KS, which was described specifically in the context of HIV infection and associated with AIDS. This form has a higher occurrence in men who have sex with men (MSM) and is more aggressive than the endemic form [277]. Iatrogenic KS is another form of KS, which is associated with immunodeficiency and develops in patients who have received immunosuppressive agents following renal transplantation, for example [278]. This form, like endemic KS, can occur with and without the presence of HIV. The fifth and final form of KS is known as HIV-negative MSM [279]. The prognosis varies depending on the subtype, ranging from indolent to highly aggressive, specifically in endemic KS.

Most manifestations of KS are similar in all subtypes. These include cutaneous and mucosal KS lesions, with the possibility of visceral involvement and lymphedema in adults and lymphadenopathy in children [274]. Disease histology is complex and highly heterogeneous. KS cells are differentiated endothelial cells that are defined by the expression of specific cellular markers like CD34 and CD36 [280]. Their morphology is best described as spindle-shaped, and they are often invaded by vasculature and lymphatic channels. The presence of LANA is used to validate the KS diagnosis.

Cellular and viral gene expression can be variable in KS, adding to the complexity of this disease [281,282]. However, in all cases, KS tumors are driven by angiogenesis, inflammation, and proliferation. The KSHV latency program is the primary driver of the lymphatic reprogramming of KS cells [283,284], which occurs through KSHV-mediated activation of the Janus kinase 2/signal transducer and activator of transcription 3 (JAK2/STAT3) and PI3K/Akt/mTOR pathways [285,286]. Activation of these pathways explains the transforming nature of KSHV infection of endothelial cells in KS. Another example is the vIL-6-mediated upregulation of hypoxia-inducible factor 1 (HIF-1), a transcription factor with pro-angiogenic properties. As previously mentioned, hypoxia has been shown to induce lytic reactivation [287]. Interestingly, KS was found to frequently occur in body extremities, where there are reduced oxygen levels. Finally, KSHV also remodels the KS tumor microenvironment by disrupting adherens junctions, resulting in increased vascular permeability [288,289]. The importance of the tumor microenvironment is further exemplified by the high dependence of KS cells on cytokines and growth factors for sustained proliferation.

### 7.2. Primary Effusion Lymphoma (PEL)

PEL is a highly aggressive type of non-Hodgkin lymphoma (NHL) that is driven by KSHV and closely associated with immuno-compromised individuals. PEL is mostly prevalent in immunodeficient populations, frequently because of HIV infection and resulting AIDS. HIV-negative patients that develop PEL are usually older men from regions around the Mediterranean Sea, where KSHV is endemic, or immunosuppressed post-transplant patients [290]. Classic PEL often forms in body cavities such as the pleural, peritoneal, and pericardial spaces. Since the discovery of PEL, a distinct but related form of PEL, known as extracavitary (EC) PEL, has been described, forming solid tumor masses [291,292].

KSHV is present in all PEL cells, with each cell containing approximately 40 to 80 copies of the KSHV genome [293]. Almost all PEL cells display the KSHV latent program of infection, with a very small percentage of cells undergoing sporadic lytic reactivation. In addition to KSHV infection, approximately 80% of all PEL display coinfection with EBV. However, the expression of EBV viral genes is highly limited, characterized by the latency 1 program, which involves the expression of EBNA1 and EBV-encoded small RNA (EBER) [294]. Although EBV does not appear to be necessary for PEL, studies have shown that EBV is important for dually infected PEL cell growth [295,296].

All PEL share similar morphologic characteristics of anaplastic, immunoblastic, or plasmablastic cells, displaying large non-cohesive cells with abnormally large nuclei [297]. More specifically, immunohistochemical analysis of patient samples shows that most PEL cells express the following markers commonly associated with hematopoietic cells: CD45, CD38, CD30, and multiple myeloma oncogene-1 (MUM1) [293,298]. The presence of these markers, along with the absence of B-cell lymphoma 6 (BCL6) and T-cell markers, indicate that PEL cells are post-germinal center B cells [299]. Unlike other viral lymphomas, PEL do not display gene translocations. Instead, the disease is driven by the anti-inflammatory and pro-survival properties of the latent proteins. The key latent players involved in PEL lymphomagenesis are LANA, vFLIP, vCyclin, vIRF3, Kaposins, and several miRNAs [51,300,301]. PEL is a rare disease with an extremely poor prognosis, characterized by a median survival rate of six months post-diagnosis [302].

### 7.3. Multicentric Castleman Disease (MCD)

Castleman disease (CD) is a group of lymphoproliferative disorders. One of these CD disorders, known as KSHV-MCD, is closely associated with KSHV infection [10]. For this review, we will only be discussing KSHV-positive MCD pathogenesis. Patients with KSHV-associated MCD frequently present with concurrent KS and are at an increased risk of developing other malignancies, such as lymphomas [303]. HIV+ patients tend to develop more aggressive cases of MCD and at a greater incidence [304]. Unlike unicentric CD, MCD is a systemic disease that is present in more than one region, defined by more than one enlarged lymph node. MCD patient sample analysis has shown that the disease cells are KSHV-infected naïve B cells that have differentiated into hyperproliferating polyclonal plasmablast cells, which express lambda light chains along with IgM heavy chains [305]. This differs from PEL cells, which have gone through the germinal center.

KSHV genes expressed during both the latent and lytic phases of the KSHV life cycle are found in MCD, including LANA, vIL-6, vIRF1, and ORF59, suggesting that both phases contribute to disease pathogenesis, which distinguishes MCD from KS and PEL [301]. Specifically, the expression of vIL-6 and its human counterpart (huIL-6) are key drivers of the proliferative nature and symptoms of MCD [305,306]. KSHV-MCD, especially in HIV+ patients, is the most aggressive form of CD. Although there are limited data regarding disease survival and prognosis, a large patient study showed that overall survival of KSHV-MCD ranged from 65% to 89% depending on HIV status [304].

### 7.4. KSHV Inflammatory Cytokine Syndrome (KICS)

KICS is a chronic syndrome that bears some similarity to KSHV-MCD. KICS occurs in patients who are positive for both HIV and KSHV. KICS differs from MCD in that it does not cause swelling of the lymph nodes; instead, it is associated with elevated levels of viral and human IL-6 as well as IL-10 [11]. Further studies have now confirmed elevated IL-10 levels and high levels of lytically replicating KSHV as biomarkers of KICS [307]. KICS frequently presents along with other KSHV-associated diseases like KS and PEL and is a very aggressive disease [308].

## 8. Therapeutics

### 8.1. Current Therapeutic Approaches

Chemotherapy remains the primary treatment option in the context of KS and PEL. For KS, chemotherapeutic regimens of bleomycin and vincristine (BV), doxorubicin, bleomycin, and vincristine (ABV), or pegylated liposomal doxorubicin (PLD) are used (Table 1) [309]. Paclitaxel, another cytotoxic agent, has been shown to have comparable effects to the use of PLD [310]. For PEL, current treatment options include regimens that apply to other types of lymphomas, and patients are treated with cyclophosphamide, doxorubicin, vincristine, and prednisone (CHOP), or etoposide, doxorubicin, cyclophosphamide, vincristine, and prednisone (EPOCH) [298]. Unfortunately, these treatments are limited and non-specific, highlighting the need for improved therapies.

A functional immune system plays a huge role in the prevention of KSHV-associated diseases. This is demonstrated by the increased prevalence of KSHV-associated diseases in HIV+ patients. Therefore, immunomodulatory therapy, like the use of highly active antiviral therapy (HAART), plays a substantial supporting role in patients who are HIV+ [311]. Ganciclovir is also an antiviral drug that has been used successfully to reduce viral replication and the risk of KS [312,313]. Of note, cidofovir, another antiviral drug, resulted in complete and sustained remission in a PEL patient, showing the potential of antiviral drug therapy [314]. However, due to the predominant latent nature of KSHV in its associated diseases, targeting viral replication has had limited efficacy.

In the context of MCD, antibody therapy targeting IL-6 (siltuximab) and IL-6 receptor (tocilizumab) as well as CD-20 (rituximab) has shown promise in patient trials [315,316,317].

**Table 1:** List of therapeutics that have been used in the context of KSHV-associated diseases in patients. The table includes information such as the name, type, target, and relevant disease in order of appearance.

Name	Type	Target	Disease	Reference
BV	Chemotherapeutic regimen	DNA/Topoisomerase II, Microtubules	KS	[309]
ABV			KS	
PLD		Topoisomerase II	KS	
CHOP		DNA/Topoisomerase II, Microtubules, Glucocorticoid receptor	PEL	[298]
EPOCH			PEL	
Paclitaxel	Chemotherapeutic agent	Microtubules	KS	[310]
Ganciclovir	Antiviral drug	DNA polymerase	KS	[312,313]
Cidofovir			PEL	[314]
Siltuximab	Antibody therapy	IL-6	MCD	[315]
Tocilizumab		IL-6 receptor	MCD	[316]
Rituximab		CD-20	MCD	[317]
Pembrolizumab and nivolumab	Immune checkpoint blockade therapy	PD-1 and PD-L1	KS	[318,319]
Nivolumab and ipilimumab		PD-1 and CTLA-4	KS	[320]
Pomalidomide and lenalidomide	Immunomodulatory imide drugs (IMiDs)	Ubiquitin E3 ligase substrate adapter Cereblon	KS/PEL	[321]
Interleukin-12 therapy	Cytokine	IL-12	KS	[322]
Rapamycin	Macrocyclic immunosuppressive drug	mTOR pathway	KS/PEL	[323,324]
Azidothymidine and interferon alpha	Nucleoside analog reverse transcriptase inhibitor (NRTI) and immunomodulator	NF-κB	PEL	[309]
Imatinib	Kinase inhibitor	Tyrosine kinase inhibitor	KS	[325]
Bortezomib	Protease inhibitor	26S proteasome	KS	[326]
Bevacizumab	Antibody therapy	VEGF inhibitor	KS	[327]

### 8.2. Targeted and Emerging Therapies

Immune checkpoint blockade therapy, specifically anti-programmed cell death protein 1 (PD1) therapy (pembrolizumab and nivolumab), has shown promise in various forms of KS [318,319]. In particular, nivolumab in combination with cytotoxic T-lymphocyte-associated protein 4 (ipilimumab) antibody therapy was effective [320]. Immunomodulatory imide drugs (IMiDs), specifically derivatives of thalidomide (pomalidomide and lenalidomide), have demonstrated clinical efficacy and low patient toxicity [321]. These drugs seem to have multifunctional lethality in KSHV-infected cells, particularly with a role in the restoration of immune surface molecules like ICAM-1 and B7-2 [328]. Furthermore, IMiDs have shown activity in patients regardless of their HIV status. Interleukin-12 therapy has also proved to be a potential antitumor agent, a cytokine that can boost the immune response and downregulate angiogenesis [322].

Other advances have been made specifically targeting pathways to which KSHV-infected cells are addicted, like the JAK/STAT, NF-κB, and PI3K/Akt/mTOR pathways. These are constitutively active and critical for cell viability both for in vitro and in vivo models and have shown promise in preclinical and early clinical studies. Rapamycin is a compound that effectively targets the mTOR pathway, providing a promising therapy specifically in the management of post-transplantation KS [324]. One study showed the efficacy of combining rapamycin with a more potent mTOR inhibitor, MLN0128, in a PEL xenograft model [323]. Inhibition of NF-κB using azidothymidine combined with interferon-alpha has proven to be highly efficient in a PEL patient [309]. Imatinib is a tyrosine kinase inhibitor that is well-tolerated and provides an alternative therapy for HIV+ patients with KS [325]. Bortezomib is a protease inhibitor that has shown promise in the same subset of patients and has proved to be well-tolerated with minimal toxicity [326]. Moreover, bevacizumab is a VEGF inhibitor that has shown activity in some KS patients [327]. An HDAC (Vorinostat) was used in KSHV+ tumors to induce lytic replication and found to be effective in combination with R-EPOCH in PEL specifically [329].

Finally, in the context of PEL, a novel therapy not involving systemic treatment has found success in HIV patients. In this study, pleurodesis was performed using bleomycin and was highly effective [330]. These novel therapeutic approaches provide a sense of hope in the treatment of KSHV-associated diseases.

### 8.3. Future Directions

Future issues that need to be addressed in the field include the lack of a vaccine preventing KSHV infection and the absence of a cure once primary infection has occurred. This is especially problematic for areas where KSHV is endemic and transmission occurs easily within families. In this regard, protease inhibitor (nelfinavir) treatment was found to result in reduced viral shedding in patients, providing a potential therapeutic approach to limiting transmission [331]. To prevent further transmission, a vaccine would be ideal.. However, vaccine strategies targeting KSHV are difficult due to the complexity of the viral life cycle and the multiple ways in which KSHV can evade the immune system. Furthermore, the limited available representative animal models have further complicated vaccine development.
